# The Effectiveness of Physiotherapy and Complementary Therapies on Voice Disorders: A Systematic Review of Randomized Controlled Trials

**DOI:** 10.3389/fmed.2017.00045

**Published:** 2017-04-24

**Authors:** Ricardo Cardoso, Rute F. Meneses, José Lumini-Oliveira

**Affiliations:** ^1^Department of Physical Medicine and Rehabilitation, Hospital-School of Fernando Pessoa University, Porto, Portugal; ^2^Transdisciplinary Center of Consciousness Studies of Fernando Pessoa University, Porto, Portugal; ^3^Fernando Pessoa University, Porto, Portugal; ^4^Hospital-School of Fernando Pessoa University, Porto, Portugal; ^5^CIAFEL – Research Centre in Physical Activity, Health and Leisure, Porto University, Porto, Portugal; ^6^LABIOMEP – Porto Biomechanics Laboratory, Porto University, Porto, Portugal

**Keywords:** voice, voice disorders, physiotherapy, complementary therapies, manual therapy, acupuncture, osteopathy, systematic review

## Abstract

The treatment of voice disorders includes physiotherapy and complementary therapies. However, research to support these treatments is scarce. Objective: to verify the effectiveness of physiotherapy and complementary therapies on voice disorders. Research on electronic databases PubMed/Medline, SciELO, and LILACS was performed using the combination: voice AND (treatment OR intervention) according to PRISMA guidelines. Only randomized controlled trials (RCTs) were included in the review. Studies were analyzed using the physiotherapy evidence database (PEDro) scale and the Center for Evidence-Based Medicine’s Levels of Evidence scale. Eight papers met the inclusion criteria. From the RCTs included in this review, six assessed massage, one transcutaneous electrical nerve stimulation (TENS), one refer to spinal manipulative therapy, and one to acupuncture. The literature regarding the effectiveness of physiotherapy and complementary therapies was good in both quality and results, indicating that massage, TENS, and acupuncture seem to be effective treatments to reduce voice complaints and improve voice quality, supporting the inclusion of complementary therapies but mostly physiotherapy interventions in the treatment of patients with voice disorders.

## Introduction

Postural changes and muscle tension have been reported in association with voice production (1, 2). Increased muscle tension around the shoulders, neck, and thorax may compromise the quality of the singing voice (3–5). This relation can be explained by the action of laryngeal mechanoreceptors, which trigger reflex adaptations in the vocal cords when stimulated by minute changes in body position (4). In this way, an appropriate muscle tone and a good posture are required for producing a good quality voice (3, 5). There are many interventions to treat voice disorders (6, 7); however, physiotherapy and complementary therapies, which improve posture and muscle tone in relation to voice have been insufficiently explored.

Postural exercises and breathing had been shown to have a positive effect on the cervical muscles and body posture, which suggests that it may improve voice quality (8) as demonstrated by the improvement of voice parameters in classical singing students by a physiotherapy program (9). Moreover, manual therapy and exercise have been shown to improve outcomes in patients with muscle tension dysphonia (10). Physiotherapy, which consisted of a systematic approach of manual therapy, education, and therapeutic exercises, had similar results to voice therapy, improving voice handicap index scores (11). Transcutaneous electrical nerve stimulation (TENS) has been shown to promote pain relief in trapezius and sternocleidomastoid muscles, decreased the root media readings in trapezius, sternocleidomastoid, and suprahyoid muscles during the production of the/e/vowel and reduce the level of dysphonia and hoarseness during spontaneous speech in dysphonic participants (12). A manual therapy, usually associated with complementary therapies like osteopathy and chiropractic but also used by physiotherapists, which that has been shown to increase range of motion, reduce muscle tension and pain which suggest that may improve voice quality (13, 14) was spinal manipulation. Another treatment which facilitated an anti-inflammatory process in phonotraumatic vocal pathologies was acupuncture (15).

To date, and as far as the authors are aware, the literature lacks a systematic review of clinical trials, showing the evidence of the effectiveness of physiotherapy and complementary therapies on voice disorders. Thus, the purpose of this systematic review is to summarize and synthesize the clinical evidence on the effectiveness of physiotherapy and complementary therapies on voice disorders.

## Methods

The systematic review was conducted according to Preferred Reporting Items for Systematic Reviews and Meta-Analyses (PRISMA) statement, whose main goal is to improve presentation patterns of systematic revisions and meta-analysis (16).

A computerized research was undertaken, by two independent reviewers, on the databases PubMed/Medline, SciELO, and LILACS in order to identify randomized controlled trials (RCTs) that evaluate the effectiveness of physiotherapy and complementary therapies on voice disorders.

The research was made according to the PRISMA flowchart and the following key words combination was used: “voice AND (treatment OR intervention).” The databases were searched from their inception through May of 2016.

The studies were included if (1) they reported interventions of physiotherapy or complementary therapies; (2) were published as full-text journal articles in English, Portuguese, French, or Spanish; (3) evaluate voice; (4) included humans; (5) had 10 or more participants; (6) included adult participants only (18 years and older); and (7) were RCTs.

The exclusion criteria were as follows: (1) literature reviews and meta-analysis; (2) case studies; (3) opinion articles; (4) assessed prevention of voice disorders; (5) used pharmacological treatments (in the same group); (6) absence of speech sample; (7) tracheostomized participants; and (8) participants diagnosed with any of the following: a voice disorder associated with local nervous system involvement (e.g., spasmodic dysphonia, vocal fold paralysis), neurological disorders (e.g., Parkinson’s or Alzheimer’s disease, amyotrophic lateral sclerosis), organic disease or trauma (e.g., keratosis, contact ulcers, papilloma’s), pediatric conditions (e.g., congenital anomalies), carcinoma, or other tumors or gastroesophageal reflux disease (17).

### Data Extraction

Two reviewers conducted the data extraction. The characteristics of the collected studies included the authors, year of publication, sample size, study design, speech sample, outcome measures, duration and type of treatment, results/conclusions, and effectiveness of treatment.

### Methodological Quality

The methodological quality of each RCT included in this review was assessed by two independent reviewers using the physiotherapy evidence database (PEDro) scoring scale (18). The PEDro scale comprises of a checklist of 11 criteria, of which only 10 criteria are scored (Table 1). For each criterion the study met, 1 point was awarded. The clear and unambiguous meeting of a criterion leads to 1 point being awarded. Consequently, a total of 10 points are available. The scale applies only to experimental studies (18). For this review, investigations with PEDro scores of 6–10 were considered high quality, of 4–5 were considered moderate quality, and of 0–3 were considered low quality (19). The PEDro scale does not evaluate clinical usefulness. The reviewers solved any rating discrepancies through verbal discussion. A consensus was reached regarding all studies during the first meeting.

**Table 1:** **Physiotherapy evidence database (PEDro) scale for measuring the study quality of randomized controlled trials**.

PEDro scoring scale [Maher et al. (18)]		
1	Eligibility criteria were specified	Yes/no
2	Subjects were randomly allocated to groups	1
3	Allocation was concealed	1
4	The groups were similar at baseline regarding the most important prognostic indicators	1
5	There was blinding of all subjects	1
6	There was blinding of all therapists who administered the therapy	1
7	There was blinding of all assessors who measured at least one key outcome	1
8	Measures of at least one key outcome were obtained from more than 85% of the subjects initially allocated to groups	1
9	All subjects for whom outcome measures were available received the treatment or control condition as allocated or, where this was not the case, data for at least one key outcome were analyzed by “intention to treat”	1
10	The results of between-group statistical comparisons are reported for at least one key outcome	1
11	The study provides both point measures and measures of variability for at least one key outcome	1

Total points		10

The Centre for Evidence-based Medicine (CEBM) Levels of Evidence scale assesses quality based on study design, which categorize the studies in a scale ranging from 1 to 5 with further subdivision for each. Systemic reviews with homogeneity of RCTs are ranked in the highest levels while expert opinions rank the least (Table 2) (20). In both scales, RCTs receive higher rankings, particularly with long-term follow-up and narrow confidence intervals.

**Table 2:** **Center of evidence-based medicine: levels of evidence**.

Level	Definition
1a	Systematic reviews of RCTs
1b	Individual RCT
1c	All-or-none studies
2a	Systematic reviews of cohort studies
2b	Individual cohort studies or low-quality RCTs
2c	Outcomes research
3a	Systematic reviews of case–control studies
3b	Individual case–control studies
4	Case series, poorly designed cohort, or case–control studies
5	Animal and bench research, expert opinion

## Results

### Studies Selection

**Figure 1:**
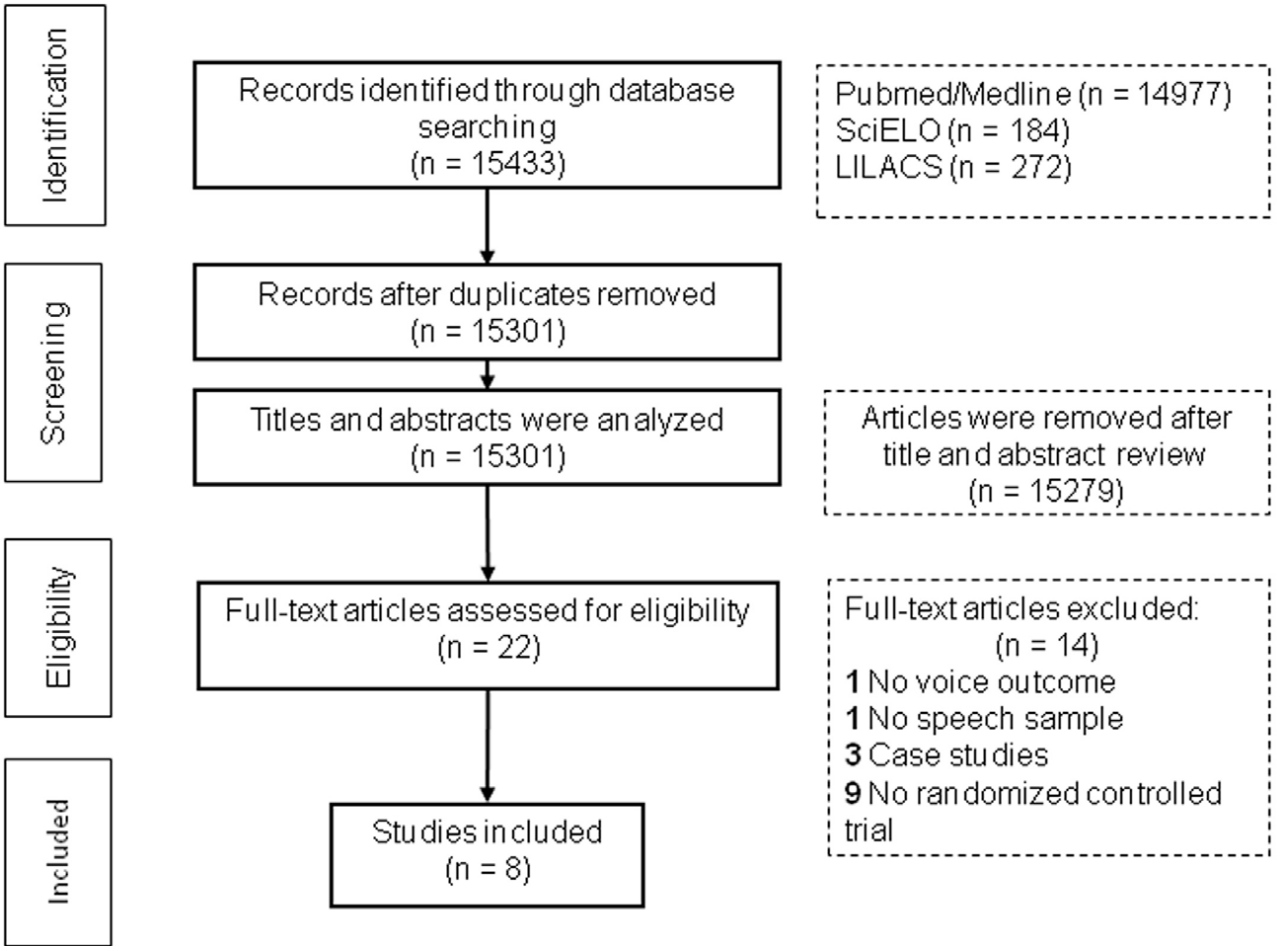
**Preferred reporting items for systematic reviews and meta-analyses flow diagram showing selection of article review**.

The literature search identified 15,433 records. After duplicates removed, 15,301 records were screened for content through title and abstract, by two independent reviewers. From these, 15,279 were excluded. The full-text of 22 articles was then assessed, by the same reviewers, for eligibility, and 14 articles were excluded. In total, eight articles were included in the systematic review. The reasons for the exclusions are listed on the PRISMA flowchart (Figure 1).

Studies were divided in four categories: massage (six studies), TENS (one study), spinal manipulative therapy (one study), and acupuncture (one study).

### Studies Description

The total number of subjects included in the 8 articles was 286, with a minimum sample size of 12 (21) and a maximum sample of 84 (22). The participants’ age ranged from 17 to 59 years. The female gender was prevalent: 225 women were included. The summary of the articles’ content is presented in Table 3.

**Table 3:** **Studies description**.

Reference	Sample size	Study design	Speech sample	Outcome measures	Duration and type of treatment	Results/conclusions	Effectiveness of treatment
Ternström et al. (23)	*N* = 31 amateur choir singers, 9 M and 22 F	RCT: parallel-group	Reading a 3-min passage of prose text	Acoustic analysis (SPL; F0) Were measured for the pre- and post-treatment recordings	Two groups: 16 in the massage group (30 min), 15 in the CG rested, lying down in silence for the same amount of time	Subjects lowered their F0 by 1.1 semitones and their SPL by 1.0 dB, with very high statistical significance. The drop in F0 was somewhat larger for the males than for the females. The control subjects showed no effect at all	↑ F0 ↑ SPL
Leppänen et al. (24)	*N* = 60 teachers, 26–57 years, m.a.: 40.6 years 0 M and 60 F	RCT: parallel-group	Sustained vowel/a/for 5 s; reading sample for 1 min at both habitual loudness and loudly	VAS (for a questionnaire about voice quality, ease or difficulty of phonation, and tiredness of the throat); perceptual-auditory analysis; acoustic analyses (F0; jitter; shimmer; Leq; α-ratio). Measurements were at the beginning and end of the autumn school term, before, and after a working day	All subjects received a 3-h VHL. After that they were divided in two groups: VM (five times in 1 h sessions; the first three sessions were given at intervals of 1 week, while the last two sessions were given at intervals of 1 month) and a VHL that received the previous 3-h VHL	The mean F0 (in reading samples) was higher and more difficulty of phonation was reported in the VHL group. Perceived pitch in loud reading increased in the VHL group and decreased in the VM group. In the VM group, the perceived firmness of loud reading decreased (*p* = 0.026). The results suggest that VM may help in sustaining vocal well-being during a school term	↑ Perceived pitch, ↑ perceived firmness of loud reading
D’haeseleer et al. (25)	*N* = 16 vocal performers, 19–25 years, m.a.: 21.4 years, 5 M and 16 F	RCT: parallel-group	Sustained vowel/a/	MPT; Acoustic analysis (F0; jitter; shimmer; noise-to-harmonic ratio); voice range profile; DSI; Self-Evaluations of Vocal Quality Questionnaire. Immediately before and after the therapy or vocal rest, an identical objective voice assessment protocol was performed	Single treatment approach. The experimental group received MCT for 20 min, whereas the CG was instructed to have complete vocal rest for 20 min	In the experimental group a significant difference in DSI was found between the measurement before and after MCT. No differences in DSI were found in the CG between the two measurements. In MCT group, improvements in MPT time and jitter were not significant	↑ DSI, = MPT, = jitter
Anhaia et al. (26)	*N* = 42 professors, 26–59 years, m.a.: 38 years, 6 M and 36 F	RCT: parallel-group	Sustained vowel/e/and an automatic speech sequence (count from 1 to 20) in regular voice	VAPP; VAS for cervical muscle tension evaluation; perceptual–auditory analysis (GRBASI); acoustic analysis (GNE; shimmer; jitter)	Two groups: perilaryngeal manual massage (G1) or vocal training (G2); 8 sessions (30 min), once a week	Both groups had an improvement in vocal symptoms. No difference in VAPP between groups. The perilaryngeal manual massage provides a slight improvement in professors’ global dysphonia level and reduces cervical tension, which is significantly reflected in self-perceived pain	G1: ↑VAPP, ↑VAS, ↑ perceptual-auditory analysis; ↑ GNE; G2: ↑VAPP, = VAS, = perceptual-auditory analysis, ↑ GNE, ↑ shimmer
Kennard, et al. (27)	*N* = 12 singers, m.a.: 24 years, 7 M and 5 F	RCT: crossover	Reading “Arthur the Rat”	Acoustic analysis (F0 and glottal closing quotient). Singers were measured acoustically immediately before and immediately after each intervention using a laryngograph. After a washout of 6 weeks, participants were switched between groups	Single treatment approach. Two osteopathic treatments: specific laryngeal manipulation (30 min) and postural manual therapy (30 min)	Positive effects of laryngeal manipulation and postural manual therapy in singers. Specific laryngeal manipulation and postural manual therapy showed a significant improvement of F0 (*p* = 0.018, *p* = 0.0143, respectively). No differences in glottal closing time	↑ F0, = glottal closing time
Fachinatto et al. (21)	*N* = 21 singers 17–51 years, m.a.: 26.3 years, 29 M and 0 F	RCT: crossover	Sing 1-min segment of a Gregorian version of the “Hail Mary” prayer	Perceptual–auditory analysis; acoustic analysis (F0; F3; F4; F5); recordings of the singing voice of each participant were taken immediately before and after the procedures. After a washout of 14 days, participants were switched between groups	Single-treatment approach. Two groups: chiropractic spinal manipulative therapy (10 min) and non-therapeutic TENS procedure (10 min)	No differences in the quality of the singing voice of asymptomatic male singers were observed on perceptual audio evaluation or acoustic analysis after a single spinal manipulative intervention of the thoracic and cervical spine	= perceptual audio, = acoustic analysis
Silvério et al. (28)	*N* = 20 w/dysphonia 18–45 years, m.a.: 21.4 years, 0 M and 20 F	RCT: parallel-group	Sustained vowel/a/isolated and after deep inspiration in pitch and usual loudness Spontaneous speech, in speed, articulation, usual pitch, and loudness, answering the questions, “What do you think of your voice?” and “Tell me about your work”	Vocal and laryngeal symptoms (questionnaire); NMSQ; VAS (pain); perceptual–auditory analysis	The volunteers were subdivided into: TENS Group (10 volunteers); LMT Group (10 volunteers). Both groups received 12 sessions of treatment, twice a week (6 weeks), lasting 20 min each	After TENS, there was significant improvement in the “high pitched voice” and “effort to speak” symptoms; there was significantly lower frequency of pain in the posterior neck and shoulder; TENS significantly reduced the intensity of pain in the posterior neck, shoulder, and upper back. The auditory perceptual analysis showed improvement only in the strain parameter after TENS. After LMT, there was improvement of the “sore throat,” significantly lower incidence of pain in the anterior neck, and the pain intensity in the posterior neck decreased. Conclusion. TENS appeared to be a treatment method intended to be used as a complement to voice therapy	After TENS: ↑ “high pitched voice,” ↑ “effort to speak,” ↑ VAS, ↑ perceptual–auditory analysis. After LMT: ↑ “sore throat,” ↑ VAS
Yiu et al. (22)	*N* = 84 w/dysphonia, 20–56 years, 18 M and 66 F	RCT: parallel-group	Sustained vowel/a/(voice range profile); sustained/i/with their tongue protruded at a comfortable pitch level for at least 5 s (laryngoscopy)	Voice range profile (maximum F0 and intensity); Size of vocal fold lesion using laryngoscopy evaluation; VAPP. Pre-treatment baseline measures were taken about an hour before the first acupuncture treatment. Second set of measures were taken 2 h after the last acupuncture session (sixth week; post-treatment), then followed by three assessments, conducted 14, 30, and 90 days after treatment. Participants in the no-treatment group were assessed on the day of enrollment, and at the 6th, 8th, 10th, and 19th week of their participation	Three groups: the genuine acupuncture group received needles puncturing nine voice-related acupoints for 30 min, two times a week for 6 weeks; the sham acupuncture group received blunted needles stimulating the skin surface of the nine acupoints for the same frequency and duration; the no-treatment group did not receive any intervention but attended the assessment sessions	Significant improvement in vocal function, as indicated by the maximum fundamental frequency produced and perceived quality of life, was found in both the genuine and sham acupuncture groups, but not in the no-treatment group. Structural (morphological) improvements were, however, only noticed in the genuine acupuncture group, which demonstrated a significant reduction in the size of the vocal fold lesions	After genuine acupuncture: ↑ voice range profile, ↑ maximum F0, ↑ VAPP, ↑ size of vocal fold lesion. After sham acupuncture: ↑ voice range profile, ↑ Maximum F0, ↑ VAPP, = size of vocal fold lesion. After no-treatment group: = voice range profile, = maximum F0, = VAPP, = size of vocal fold lesion

*Code—CG, control group; Leq, equivalent sound level; F5, fifth formant; F4, fourth formant; F0, fundamental frequency; GNE, glottal noise energy; GRBASI, grade, roughness, breathiness, asteny, strain, instability; ↑, improvement; LMT, laryngeal manual therapy; MCT, manual circumlaryngeal therapy; MPT, maximum phonation time; m.a., mean age; MTD, muscle tension dysphonia; MPQ, musculoskeletal pain questionnaire; =, no changes; NMSQ, nordic musculoskeletal symptoms questionnaire; RCT, randomized controlled trial; SNR, signal-to-noise ratio; SPL, sound pressure level; F3, third formant; TENS, transcutaneous electrical nerve stimulation; VAS, visual analog scale; VAPP, voice activity and participation profile; voice hygiene lecture; VM, Voice MassageTM; w/, with*.

The studies presented some homogeneity regarding speech samples, since sustained vowel was present in five articles (22, 24–26, 28), followed by reading (23, 24, 27), spontaneous speech (28), automatic speech sequence (26) and singing (21).

Taking into account the voice assessment parameters, the investigations were similar because of the eight RCTs, six used acoustic analysis (21, 23–27), and four included perceptive–auditory analysis (21, 24, 26, 28). However, regarding other outcome measures, the studies were heterogeneous varying from voice range profile (22, 25), maximum phonation time (MPT) (25), size of vocal fold lesion (22), muscle palpation (26), visual analog scale (VAS) (24, 26, 28), and scales like voice activity and participation profile (VAPP) (22, 26) and nordic musculoskeletal symptoms questionnaire (28).

Most of the studies had two groups, where a single experimental group was compared to a control group (CG) (23–25), or to two different interventions (26–28) or where an experimental group was assessed versus a placebo group (21). However, one investigation (22) had three groups (intervention group, sham intervention group, and no-treatment group). In terms of study design, six studies were RCTs (parallel-group) (22–26, 28), and two were RCTs (Crossover) (21, 27).

Most interventions had a duration of 30 min (22, 23, 26, 27). However, some were 10 min (21), 20 min (25, 28), or 60 min (24) long. The length of the studies varied from a single treatment approach (21, 23, 25, 27) to 6 weeks (22, 28), 8 weeks (26), or 3 months of intervention (24).

The studies presented some heterogeneity regarding the professional background of the therapists who administered the therapy and in the years of experience. The professional background of the intervener in the articles that do not mention the years of experience range from therapist (28), physiotherapist (21, 26), chiropractor (21), osteopath (27), to a naprapathy therapist (23). When the experience is mentioned, it varies from acupuncturists (22) with 2 years of experience, to experienced voice therapists (25) or voice massage therapists with 10 years of experience (24).

All studies showed positive results in their interventions, with exception of the spinal manipulative therapy (21).

### Studies Quality and Levels of Evidence

The mean PEDro score for the studies included in the review was 8.25 points (range: 7–10 points) (Table 4).

**Table 4:** **Methodological quality [physiotherapy evidence database (PEDro) scale] and levels of evidence [centre for evidence-based medicine (CEBM)] of included studies**.

Reference	Present criteria on PEDro scale	Total score on PEDro scale	Level of evidence (CEBM)
Ternström et al. (23)	2, 3, 4, 5, 8, 9, 10, 11	8/10	2b
Leppänen et al. (24)	2, 3, 4, 5, 7, 8, 9, 10, 11	9/10	1b
D’haeseleer et al. (25)	2, 3, 4, 5, 8, 9, 10, 11	8/10	2b
Anhaia et al. (26)	2, 3, 4, 8, 9, 10, 11	7/10	2b
Kennard et al. (27)	2, 3, 4, 8, 9, 10, 11	7/10	2b
Fachinatto et al. (21)	2, 3, 4, 5, 6, 7, 8, 10, 11	10/10	2b
Silvério et al. (28)	2, 3, 4, 7, 8, 9, 10, 11	8/10	2b
Yiu et al. (22)	2, 3, 4, 5, 7, 8, 9, 10, 11	9/10	1b

According to the quality criteria set, the average quality of the studies included in this review is high.

There was not a high degree of variation in quality between studies; however, only one study (21) was able to satisfy the blinding criteria for the therapists (PEDro scale question 6), four studies (23, 25–27) were not able to satisfy the blinding criteria for the assessors (PEDro scale question 7), and three RCTs (26–28) were not able to satisfy the blinding criteria for the subjects (PEDro scale question 5). The remaining criteria were always scored positively. Given the difficulty to comply with the blinding criteria given the nature of the intervention performed, it is unsurprising that there were difficulties to apply it. Only one trial was able to meet all the criteria (21).

Having regard to evidence levels, only two studies rated as 1b (22, 24) and six studies as 2b (21, 23, 25–28) in the CEBM ratings. The most common reason for a 2b rank was that the study had a small sample size.

### Massage

Massage appears to improve vocal quality, muscle tension, and pain through VAS and VAPP. The average quality of the studies included in this section of the review was slightly lower than the average quality of the studies in the overall review (mean PEDro score = 7.83; range: 7–9). In terms of levels of evidence, one study was rated as 1b and the others were rated as 2b. To date, six RCTs have explored the effects of massage on voice. All of them showed good results.

In the investigation of Ternström et al. (23), 31 amateur choir singers performed a 30 min massage. They found that massage improved fundamental frequency (F0) and sound pressure level. D’haeseleer et al. (25) conducted a study in 16 vocal performers where the experimental group received a single treatment approach of manual circumlaryngeal therapy (MCT) for 20 min, whereas the CG was instructed to have complete vocal rest for 20 min. In the experimental group, a significant difference in dysphonia severity index was found before and after MCT. No differences in dysphonia severity index were found in the CG between the two measurements. In the MCT group, improvements in MPT and jitter were not significant. In another study (26), 42 professors were divided into two groups: perilaryngeal manual massage or vocal training. Participants performed eight sessions (30 min), once a week. The authors found that both groups had an improvement in vocal symptoms. No difference was verified in VAPP between groups. The perilaryngeal manual massage provided a slight improvement in professors’ global dysphonia level and reduced cervical tension, which was significantly reflected in the self-perceived pain score. Silvério et al. (28) demonstrated that laryngeal manual therapy (LMT) promoted an improvement of the “sore throat,” significantly lowering the incidence of pain in the anterior neck, and posterior neck. Twenty dysphonic participants received 12 sessions of treatment, twice a week (6 weeks), lasting 20 min each. Kennard et al. (27) conducted a crossover RCT where 12 asymptomatic singers received specific laryngeal manipulation (30 min) or postural manual therapy (30 min). Both groups showed a significant improvement of F0 (*p* = 0.018, *p* = 0.0143, respectively). No differences were found in glottal closing time. The study of Leppänen et al. (24) was rated as a high-quality one, ranked at level 1b on the CEBM scale and earned 9 of 10 points on the PEDro scale. The 1b rating reflects a study that was well designed, with a sufficient number of participants and adequate long-term follow-up. The PEDro score indicates that the study design was strong. All 60 teachers received a 3-h voice hygiene lecture (VHL). After that they were divided into two groups: Voice Massage™ (VM) group (five times in 1 h sessions; the first three sessions were given at intervals of 1 week, while the last two sessions were given at intervals of 1 month) and a VHL group that received the previous 3-h VHL. Measurements were at the beginning and end of the autumn school term, before and after a working day. The mean F0 (in reading samples) was higher and more difficulty of phonation was reported in the VHL group. Perceived pitch in loud reading increased in the VHL group and decreased in the VM group. In the VM group, the perceived firmness of loud reading decreased (*p* = 0.026). The results suggest that VM may help in sustaining vocal well-being during a school term.

### Transcutaneous Electrical Nerve Stimulation

Only one RCT (28) was included in this section. The quality of the study was high (PEDro score = 8) and was rated as 2b in terms of levels of evidence. Authors concluded that TENS can reduce the intensity and frequency of pain in shoulders, upper back, and neck. They also found that TENS group had symptoms that significantly improved from “thin voice” and “effort to speak.” From the analysis of the vowel/a/, they verified that 60% of the participants in TENS group significantly improved the “tension” parameter in voice, which did not occur with the women of the LMT group. In the analysis of spontaneous speech, no significant differences were found in all of the parameters analyzed. In this study, 20 dysphonic participants received a 12 sessions of treatment, twice a week (6 weeks), lasting 20 min each. The TENS application consisted the bilateral placement of electrodes (5.0 × 5.0 cm) on the upper trapezius region and in the submandibular region, with a low (10 Hz), symmetrical biphasic square pulse, phase 200 ms, at a motor threshold intensity. According to the authors (28), TENS applied in this way can promote muscle relaxation because stimulates both nociceptive fibers type A-delta and C and motor efferent fibers, producing not just an analgesic effect, but also decreasing the symptoms in the vocal tract.

### Spinal Manipulative Therapy

Spinal manipulative therapy showed no differences in the quality of the singing voice of asymptomatic male singers, when compared to non-therapeutic TENS procedure. The quality of the study (21) was the highest in this review (PEDro score = 10) and was rated as 2b in terms of levels of evidence. Comparing to CG, authors found no differences on perceptual audio evaluation or acoustic analysis of the male singers, after a single spinal manipulative intervention of the thoracic and cervical spine. According to researchers (21), the parameters used for non-therapeutic TENS application influenced participants psychologically and provided a certain degree of muscle relaxation, consequently improving voice quality.

### Acupuncture

Yiu et al. (22) conducted a high-quality study that was rated at level 1b on the CEBM scale and earned 9 of 10 points on the PEDro scale. In this RCT, 84 participants with dysphonia were divided in three groups: genuine acupuncture group that received needles in nine voice-related acupoints (two Hegu and two Lieque points at the wrists, one Lianquan, and two Renying points in the neck, and two Zhaohai points at the ankles). Disposable stainless steel needles (0.25 mm diameter, 25 mm long with a 25 mm extended handle) were used, with a depth about 13 mm for Zhaohai, Hegu, and Lianquan and 5–8 mm for Lieque and Renyin acupoints. Stimulation was applied by twirling the needles once every 5 min for 30 min; the sham acupuncture group received blunted needles stimulating the skin surface of the nine acupoints for the same frequency and duration as the genuine acupuncture group; and the no-treatment group. A significant improvement in vocal function, as indicated by the maximum fundamental frequency produced was verified in both the genuine and sham acupuncture groups, but not in the no-treatment group. About perceived quality of life, genuine acupuncture groups showed significant results comparing to sham acupuncture group (*p* = 0.003) and no-treatment group (*p* = 0.01). No significant difference was found between the no-treatment and sham acupuncture group (*p* = 0.83). Only the genuine acupuncture group demonstrated a significant reduction in the size of the vocal fold lesions.

## Discussion

Voice disorders are a common problem that occurs more frequently in teachers than in other professions that use the voice frequently (29–31). The results of the present review support this notion since teachers were the most prevalent professionals. The predominance of female participants in this study (225 out of 286 participants) is a consistent feature in the international literature, as the female gender is predominant in the teaching profession and having a higher prevalence of vocal problems, due to the demand of the profession (32–34).

Individuals with functional or organofunctional dysphonia may have an increase in muscle tension in cervical and perilaryngeal muscles (35, 36), muscle pain at rest or during function (36, 37), reduced range of motion in cervical spine (36, 37), hyperactivity of extrinsic laryngeal muscles (37), and postural changes (36).

A proven effective method to induce normotension of cervical and laryngeal muscles is manual therapy, such as laryngeal massage and massage on the neck or shoulder girdle, as well as passive stretch. Manual therapy has been used by professionals from several different fields, such as speech-language therapists (23, 26), osteopaths (27), and physiotherapists (9–11). This variety reflects different descriptors or denominations that are used in research and clinically. The main goal of this technique is to relax the muscles that are excessively tense, which ends up inhibiting the balance of phonatory function. This may enlarge the diameter of the resonance cavity, particularly at the pharynx, which is an important goal of classical singing (38). The high position of the larynx may influence the vocal function, changing length control and the rigidity of vocal folds, which contributes to the decrease in the vocal quality (3, 5).

Manual therapy, in the form of massage, was the intervention most frequently used in the studies reviewed, being present in six of the eight articles. This treatment was effective in all studies. It should be noted that massage was helpful in sustaining vocal well-being during a school term (24).

Another technique of manual therapy is spinal manipulative therapy. The authors (21) that conducted this investigation found no differences in the voice quality. Once excessive cervical muscle tension was not a criterion for subject selection, it is possible that most participants did not have excessive muscle tension and, therefore, spinal manipulation was not effective on the singing voice. Moreover, spinal manipulation cannot be a standardized intervention as it was used, as depends on the origin of the dysfunction. So, in cases of postural misalignment, where the head and neck are not properly aligned, spinal manipulation may improve posture, especially the positioning of the head relative to the thorax and reduce cervical and thoracic muscle tension, thus reducing tension during sound emission and increasing pharyngeal cavity volume (1, 4, 5, 13, 14). Reducing muscle tension and improving posture to have better voice quality are corroborated in the studies of Craig et al. (11), Tomlinson and Archer (10), and Staes et al. (9), where they successfully applied physiotherapy programs to improve posture, pain, and voice quality. These programs consist of spinal manipulation (9), joint mobilizations (11), myofascial release (11) contract-relax techniques (10, 11), cervical stretches (10), therapeutic exercises of neck (9, 11) and lumbar spine (9, 11), pelvic floor exercises (9), ergonomics (10, 11), and breathing exercises (9).

In this review, one RCT (28) about another usual physiotherapy treatment, TENS was included showing good results. An investigation with similar results was conducted by Guirro et al. (12), where 10 participants with dysphonia performed 10 TENS sessions (200 µs and 10 Hz; 4 electrodes applied in the trapezius and sternocleidomastoid muscles bilaterally), with pain relief through VAS. The TENS decreased the Root Media readings of right and left trapezius, left sternocleidomastoid, and suprahyoid muscles during the production of the/e/vowel and for right and left trapezius as well as right and left sternocleidomastoid during spontaneous speech. The voice analysis showed a decrease in the level of laryngeal injuries; with no difference during the production of the/e/vowel in the perceptive–auditory analysis and with a decrease in the level of dysphonia and hoarseness during spontaneous speech.

The other treatment included in this review was acupuncture, a method of complementary medicine (39). The RCT included (22) demonstrated that acupuncture was more effective than a CG receiving no treatment or a sham treatment. In other investigation, Yiu et al. (14) showed that acupuncture of voice-related acupoints facilitated an anti-inflammatory process in phonotraumatic vocal pathologies of 17 dysphonic individuals.

The experience and training of the therapists who gave the treatments were mentioned in very few studies. No adverse events were reported in the trials included in this review.

The methodological quality of the included RCTs was high. However, only one study was able to satisfy the blinding criteria for the therapists, four studies were not able to satisfy the blinding criteria for the assessors, and three RCTs were not able to satisfy the blinding criteria for the subjects.

Regarding the evidence levels, only two studies rated as 1b and six studies as 2b in the CEBM ratings, demonstrating an overall good quality for the studies.

The results of the studies were encouraging. There is evidence that massage, TENS, and acupuncture may be useful as either a unique therapy or as an adjunct therapy to other established treatments for voice disorders.

This review may be subject to several critics. Some studies had small samples and did not evaluate physical parameters like posture or muscle tension. Most of them had short follow-ups, with exception of one study (22) that performed repeated assessments 14, 30, and 90 days after baseline. Most of the studies had a short duration, since many of them had a single treatment approach and some of them do not refer the experience of the therapists who administered the therapy.

Despite these limitations, this review is, as far as the authors are aware, the first to comprehensively and critically assess evidence of the effectiveness of physiotherapy and complementary therapies on voice disorders, which may provide useful insight for further studies. This review can be considered as a first step in order to document physiotherapy and complementary therapies approaches in dysphonic participants, singers, and teachers, with the aim to improve postural alignment and optimize muscle activity.

To attain the highest-quality evidences, good quality RCT designs should be utilized in the future researches. Participants should be randomized, the design should be double blinded, and the clinician performing the treatment should use it regularly in clinical practice. As well, if possible, treatments should be compared with a control (no-treatment) group, placebo group, and with other established treatments. We also suggest further RCTs with healthy participants (especially teachers and singers) and with individuals with voice or cervical complaints, larger samples, and long-term follow-ups, in order to verify long-term effects. Posture evaluations, electromyography, and muscle palpation are needed to improve assessment protocols as well as the combination of physiotherapy or complementary therapies with voice therapy programs. These guidelines will result in higher-quality studies that can help us determine the true effectiveness of these therapies as a treatment for voice disorders.

## Conclusion

The literature regarding the effectiveness of physiotherapy and complementary therapies was good in both quality and results. The evidence from the studies included in the review suggest that manual therapy through laryngeal massage and massage of the neck or shoulder girdle is an effective treatment to reduce dysphonia complaints and muscle tension and to improve voice quality. It is important to emphasize that the TENS and acupuncture also presented good results. The knowledge of the relationship between body posture, laryngeal muscles, voice production, and dysphonia is of paramount importance because a transdisciplinary action can optimize evaluation and treatment in order to provide clinically significant benefits to patients with voice problems.

## Author Contributions

RC conceived the study. RM and JL-O supervised and oriented all aspects of its implementation, including its planning.

## Conflict of Interest Statement

The authors declare that the research was conducted in the absence of any commercial or financial relationships that could be construed as a potential conflict of interest.
